# A Proximity Mapping Journey into the Biology of the Mammalian Centrosome/Cilium Complex

**DOI:** 10.3390/cells9061390

**Published:** 2020-06-03

**Authors:** Melis Dilara Arslanhan, Dila Gulensoy, Elif Nur Firat-Karalar

**Affiliations:** Department of Molecular Biology and Genetics, Koc University, 34450 Istanbul, Turkey; marslanhan18@ku.edu.tr (M.D.A.); dgulensoy15@ku.edu.tr (D.G.)

**Keywords:** centriolar satellites, centrosome, cilia, microtubules, ciliopathies, proximity-labeling, BioID, APEX, TurboID

## Abstract

The mammalian centrosome/cilium complex is composed of the centrosome, the primary cilium and the centriolar satellites, which together regulate cell polarity, signaling, proliferation and motility in cells and thereby development and homeostasis in organisms. Accordingly, deregulation of its structure and functions is implicated in various human diseases including cancer, developmental disorders and neurodegenerative diseases. To better understand these disease connections, the molecular underpinnings of the assembly, maintenance and dynamic adaptations of the centrosome/cilium complex need to be uncovered with exquisite detail. Application of proximity-based labeling methods to the centrosome/cilium complex generated spatial and temporal interaction maps for its components and provided key insights into these questions. In this review, we first describe the structure and cell cycle-linked regulation of the centrosome/cilium complex. Next, we explain the inherent biochemical and temporal limitations in probing the structure and function of the centrosome/cilium complex and describe how proximity-based labeling approaches have addressed them. Finally, we explore current insights into the knowledge we gained from the proximity mapping studies as it pertains to centrosome and cilium biogenesis and systematic characterization of the centrosome, cilium and centriolar satellite interactomes.

## 1. Structure of the Mammalian Centrosome/Cilium Complex

The mammalian centrosome/cilium complex is composed of the centrosome, the primary cilium and the centriolar satellites [1,2,3]. Proper assembly and functions of the centrosome/cilium complex require highly regulated, dynamic communication between its three compartments. In this section, we first describe the morphological, structural and functional features of the centrosomes, the primary cilium and the centriolar satellites in detail, which will set the stage for discussing the inherent limitations in defining their components and interactions in the subsequent section.

### 1.1. Centrosomes

**Figure 1:**
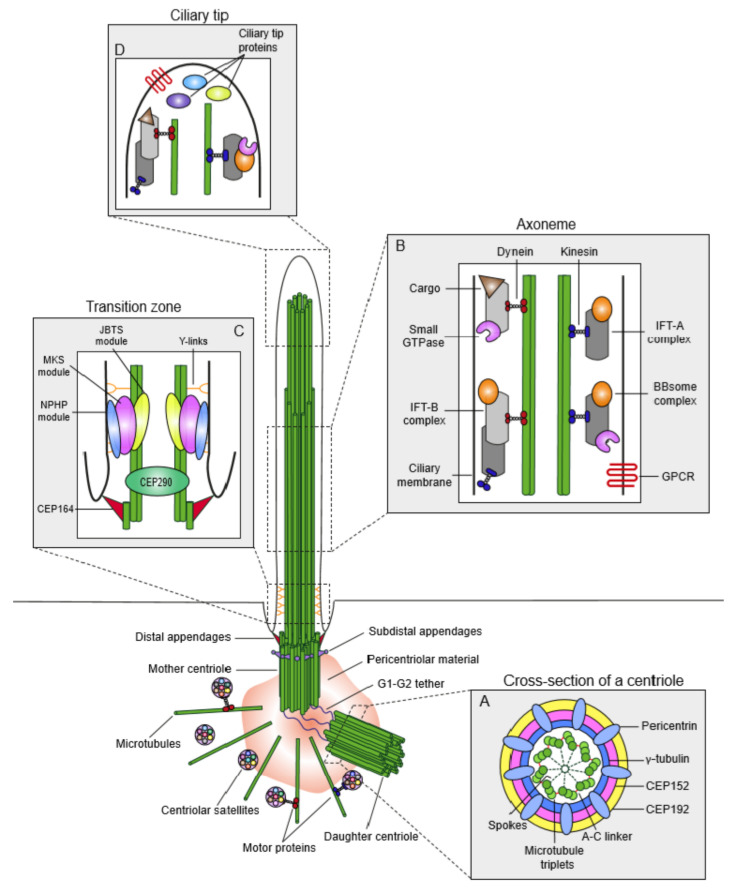
Overview of the anatomy of the centrosome/cilium complex and its sub-compartments. The centrosome/cilium complex is composed of the centrosome, the primary cilium and the centriolar satellites. At the core of the centrosome are two microtubule-based barrel-shaped centrioles, which recruit pericentriolar material (PCM). PCM contains gamma-tubulin ring complexes and functions in microtubule nucleation and organization. In interphase cells, centrioles are tethered to each other by the filamentous structure termed the “G1-G2 tether”. The two centrioles of the centrosome differ in age, structure and maturity. The older centriole is called the mother centriole and the younger centriole is called the daughter centriole. Mother centriole harbors distal and subdistal appendages at its distal end and functions as the basal body to template primary cilium assembly. The primary cilium is compartmentalized into structurally and functionally distinct regions, which include the transition zone, the ciliary axoneme and the ciliary tip. Centriolar satellites are membrane-less granules that cluster around the centrosome. They exhibit microtubule- and molecular motor-dependent active motility as well as Brownian diffusion. (**A**) Cross-section of the proximal end of the centrioles. Centriole barrel contains symmetrically arranged nine microtubule triplets connected by A–C linkers. The microtubule triplets are connected to the inner core by radial spokes. The inner core is a helical scaffold of a dense matrix that provides structural integrity and flexibility to the centriole barrel. PCM is organized into concentric layers of proteins that are spanned by radially extended filamentous structures formed by CEP152 and pericentrin. (**B**) The transition zone connects the outer microtubule doublets to the plasma membrane and functions as the diffusion barrier that regulates protein entry into and exit out of the primary cilium. The transition zone is composed of the NPHP-MKS-JBTS module. Transition fibers are the distal appendages of the basal body, which anchor the basal body to the ciliary membrane. In fact, transition fibers correspond to the distal appendages of the mother centriole. (**C**) The microtubule-based ciliary axoneme forms the core of the primary cilium and serves as tracks for ciliary transport complexes including the IFT-A, IFT-B and BBSome. The anterograde movement of the IFT-B complex and the retrograde movement of the IFT-A complex is powered by molecular motors kinesin-2 (blue) and cytoplasmic dynein-2 (red). BBSome complex interacts with IFT particles and mediates removal of GPCRs from the cilium. (**D**) The ciliary tip is the specialized region at the distal end of the cilium, which contains IFT particles, Hedgehog pathway components and microtubule-associated proteins and regulates IFT remodeling, cilium length and Hedgehog signaling.

Centrosomes function as the main microtubule-organizing centers (MTOC) of animal cells and play critical roles in a wide range of cellular processes such as cell polarity, adhesion, motility and division. Unlike most organelles, centrosomes are not bounded by membranes, instead, they are structurally organized, dynamic macromolecular protein complexes [4]. Each centrosome is composed of two orthogonally-positioned centrioles surrounded by an electron-dense matrix termed the pericentriolar material (PCM) (Figure 1). PCM serves as a scaffold to concentrate proteins required for microtubule nucleation and organization as well as cellular signaling [5,6]. Centrioles are barrel-shaped, microtubule-based structures conserved from ciliated protists to humans [7,8]. While the centriole size varies across different organisms and within different cell types of the same organism, all centrioles are characterized by a nine-fold symmetry and proximal-distal polarity [9,10,11]. In vertebrates, nine compound microtubules are arranged circumferentially to assemble the centriole barrel [12]. The proximal 60% of the human centrioles consists of triplet microtubules, which contain a complete inner A-tubule onto which partial B and C tubules are assembled (Figure 1A) [12,13]. The remaining distal part instead has nine doublet microtubules. Notably, the microtubule scaffold of the centriole confers them an inherent polarity, in which the minus ends define the proximal end and plus ends define the distal end. Depending on their maturity stage and species, centrioles harbor appendages at their distal end and the cartwheel at their proximal end. The region between proximal and distal ends is the central core, which contains Y-shaped linkers [14,15].

**Figure 2:**
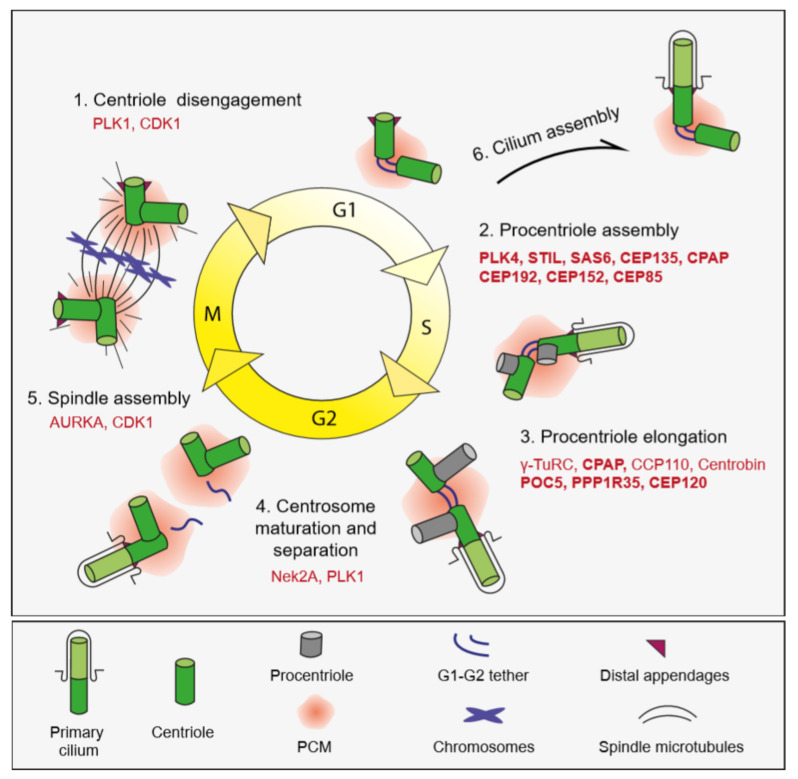
Regulation of the centriole and cilium biogenesis during the cell cycle. Centriole and cilium biogenesis are highly regulated, multi-step processes that are tightly linked to the cell cycle. In the G1 phase, the majority of animal cells have one centrosome composed of a pair of centrioles tethered by the G1-G2 tether at their proximal ends. At the G1 and S phases the two centrioles duplicate only once such that one procentriole forms adjacent to each pre-existing parental centriole. This step is governed by the sequential centriolar recruitment and activity of a conserved set of proteins including the kinase PLK4, the scaffold STIL and the building block of the cartwheel SASS6 along with regulators of these proteins. Following initiation of centriole duplication, procentrioles elongate throughout S and G2 phases. In late G2, the two centrosomes are separated by the dissolution of the G1-G2 tether. In a process termed centriole-to-centrosome conversion, fully elongated centrioles lose their cartwheel and recruit more PCM material in preparation for bipolar spindle assembly. During mitosis, centrosomes assemble the bipolar spindle, which equally segregates both a pair of centrioles and genetic material to daughter cells. Distal appendages undergo transient disassembly during mitosis. At the end of mitosis, the centriole pairs disengage and lose their orthogonal arrangement. Centriole disengagement relicenses the centrioles for centriole duplication in the next cell cycle. As cells enter quiescence by depletion of growth factors, cilium assembles. Steady-state cilium persists into S/G2/M phases and completes disassembly close to cytokinesis after nuclear envelope breakdown.

In interphase, the two centrioles of the centrosome are tethered to each other at their proximal ends by a loose proteinaceous linker called the “G1-G2 tether”, which enables centrosomes to function as a single microtubule-organizing center (Figure 2) [16,17,18]. During G2/M transition, phosphorylation and subsequent loss of the G1-G2 tether induces separation of centrosomes, which is required for the assembly of the bipolar spindle and segregation of chromosomes in mitosis [19]. At the beginning of the S phase, centriole duplication is initiated by the formation of a procentriole adjacent to a pre-existing centriole, to which it is connected by the “S-M linker” (Figure 2) [18]. Following cartwheel assembly, the procentriole elongates into full-length centrioles throughout S and G2 phases. In a process called centriole-to-centrosome conversion, full-length centrioles lose their cartwheel, recruit PCM and disengage from the parental centrioles by dissolution of the S-M linker and acquire PCM [20]. The centriole completes its maturation by acquiring appendages in the next cell cycle.

As a result of the centriole duplication cycle, the two centrioles of the centrosome differ in age and maturity and thereby they have different functions (Figure 1) [18]. The younger centriole that assembled in the previous cell cycle is called the “daughter” centriole. The older centriole is called the “mother” centriole. The distal end of the mother centriole harbors two distinct appendage structures—distal appendages, which are organized in a nine-fold symmetrical structure and are required for centriole docking to the membrane during ciliogenesis, and subdistal appendages, which exist in variable numbers and act as an anchor for a subset of cytoplasmic microtubules [21,22].

Recent advances in super-resolution microscopy and cryo-electron tomography have revealed unprecedented insight into the architecture and composition of centrioles. In particular, they enabled assignment of the centriole proteins to distinct centriolar sub-regions such as the centriole inner core and appendages [14,15,23,24,25]. Centrioles are not just hollow, empty barrels; instead, about 70% of their lumen contains an inner helical scaffold that forms a dense lattice by interconnecting the microtubule triplets [15,26]. Recent studies suggested structural functions for the inner core in the maintenance of centriole microtubule triplet cohesion and geometry [15,27]. Application of Ultrastructure Expansion Microscopy (U-ExM) to the centriole inner core identified and mapped POC5, POC1B, Centrin-2, FAM161A and WDR90 as the five components of a microtubule-associated complex associated with the helical scaffold [27]. In addition to the inner core, the molecular and structural code of the distal appendages were also unraveled by with nanoscale precision [23,25,28]. The 3D localization map of distal appendages was generated by combining Stochastic Optical Reconstruction Microscopy (STORM) with 2D and 3D EM analysis [23]. This map revealed the presence of a filamentous base that mediates the association of distal appendages with two microtubule triplets of the centriole. Finally, super-resolution imaging and functional characterization of cells genetically deleted for different distal and subdistal appendage proteins revealed a hierarchical assembly pathway for both distal and subdistal appendages [23,29,30,31].

The proximal end of the centrioles recruits PCM to form the centrosome. PCM nucleates, anchors and releases microtubules and confers the centrosome its microtubule-organizing center activity [6,32]. Additionally, PCM has recently emerged as a signaling center due to its scaffolding activity in concentrating signaling components such as kinases and phosphatases [5]. At the ultrastructural level, PCM had long been considered as an amorphous structure. However, its high-resolution structure revealed by super-resolution microscopy changed this paradigm and unraveled a remarkable degree of organization within the PCM [33,34,35,36,37]. In interphase cells, the majority of the PCM components studied are organized in concentric rings around the centriole. Exceptions are pericentrin and Cep152, which extend radially from the centriole with their C-termini adjacent to the centriole wall and N-termini facing outward (Figure 1A) [34,37,38]. As cells progress into mitosis, PCM expands by recruitment of more components and increases its microtubule nucleation capacity in preparation for mitotic spindle formation. This process, termed “centrosome maturation”, requires the cooperative activity of multiple mitotic kinases including Polo-like kinase 1 (PLK1) and Aurora A [39,40,41]. During the mitotic expansion of PCM, proteins accumulate in a cloud-like matrix and thus mitotic PCM is less ordered than interphase PCM [42].

### 1.2. Primary Cilium

Cilia and flagella are microtubule-based extensions that protrude from the cell surface and play critical roles during cellular signaling (i.e., primary cilium), cell motility (i.e., flagella of sperm cells) and movement of extracellular fluid (i.e., motile cilia of tracheal epithelial cells). In this review, we discuss only the primary cilium as part of the mammalian centrosome/cilium complex and readers are invited to consult to recent reviews on other cilia types [43,44,45]. Primary cilium is a non-motile sensory organelle that functions as a nexus for signaling pathways important for development and tissue homeostasis [46,47]. Accordingly, defects in its structure and function cause multisystemic developmental disorders termed sensory ciliopathies, which are characterized by retinal degeneration, polycystic kidney disease, obesity and polydactyly among others [48].

The primary cilium is compartmentalized into structurally and functionally distinct regions (Figure 1). At the core of the primary cilium is the microtubule-based axoneme, which is surrounded by the ciliary membrane (Figure 1B). In quiescent cells, the mother centriole acts as the basal body to template the assembly of the axoneme [49,50]. The basal body is anchored to the plasma membrane through its distal appendages. Recent electron tomography studies of the cilium generated its 3D structural maps, which significantly changed the widely accepted 9+0 paradigm for the axonemal organization of ciliary microtubules [51,52]. According to the revised model, the nine radially arranged microtubule doublets at the proximal end of the axoneme transitions into an unstructured bundle of microtubule singlets of variable lengths towards the distal end of the cilium. Of note, the microtubule singlet region occupies the majority of the axoneme. The distal end of the cilium between the ends of microtubule singlets and the ciliary membrane is the “ciliary tip”, which is critical for IFT remodeling, regulation of cilium length and Hedgehog signaling (Figure 1D) [53].

In addition to providing structural rigidity and flexibility to the cilium, the ciliary axoneme serves as a track for ciliary transport complexes that mediate protein trafficking into and out of the cilium [54,55]. Assembly and maintenance of the cilium mainly depend on the highly conserved intraflagellar transport (IFT) complexes, which are composed of the anterograde IFT-B and retrograde IFT-A complex (Figure 1B). IFT complexes interact with ciliary cargo and bidirectionally move within the cilium, which is powered by the plus-end directed kinesin-2 and minus-end directed dynein-2 motors [56]. Among the cargoes trafficked by IFT are structural components of the axoneme such as tubulin dimers and signaling proteins such as ciliary membrane receptors. In addition to IFT, the BBSome complex is the other conserved ciliary transport machine, which is composed of eight Bardet–Biedl syndrome (BBS) proteins. The BBSome complex mediates removal of G protein-coupled receptors (GPCRs) from the cilium by undergoing retrograde transport in association with the IFT-A complex (Figure 1B) [57].

The most proximal region of the cilium is the ciliary gate, which consists of the transition fibers and the transition zone (Figure 1C) [58,59]. The transition fibers anchor the basal body to the ciliary membrane and function as docking sites for IFT complexes. In fact, transition fibers correspond to the distal appendages of the mother centriole. The transition zone is characterized by Y-shaped linkers (Y-links) that connect the outer microtubule doublets to the plasma membrane and the ciliary necklace that consists of circumferential strands of intramembrane particles. The transition zone functions as the “diffusion barrier” to regulate selective ciliary protein entry and exit. Consistent with its critical functions in cilium content regulation, many proteins that are mutated in ciliopathies are components of the transition zone. Genetic interaction, super-resolution imaging and proteomics studies together identified three transition zone protein modules, which include Meckel–Gruber Syndrome (MKS), nephronophthisis (NPHP) and Joubert syndrome (JBTS) modules (Figure 1C) [60]. These modules cooperate to assemble and maintain a functional transition zone. 

Primary cilium biogenesis is a highly regulated, multistep process that is initiated when cells exit the cell cycle in response to mitogen deprivation or differentiation cues [1,49,61]. Ultrastructural studies provided a detailed description of the steps of cilium assembly in different cell types and showed that there are two distinct ciliogenesis pathways [62,63]. In the extracellular pathway, used by lung and kidney epithelial cells, the mother centriole docks to the plasma membrane via distal appendages and directly initiates the assembly of the ciliary axoneme that protrudes into the extracellular environment [64]. The intracellular pathway was described in fibroblasts, smooth muscle cells and retinal epithelial cells [62,64]. It starts with the recruitment and docking of Golgi-derived periciliary vesicles (PCVs) at the distal appendages of the mother centriole [65]. After the fusion PCVs into a larger ciliary vesicle (CV), the axonemal microtubules elongate and transition zone forms, which together give rise to a nascent ciliary structure within the cytoplasm. At this stage, the axoneme is surrounded by an inner membrane that eventually becomes the ciliary membrane and the outer membrane called the “sheath” [64]. The fusion of the sheath with the plasma membrane exposes the cilium to the extracellular environment, which continues to elongate to assemble the mature cilium. Specific to the intracellular cilia is the ciliary pocket, which is formed by the invagination of the plasma membrane adjacent to the ciliary membrane. The ciliary pocket has been proposed to act as a platform to mediate ciliary endocytic activity and vesicular trafficking as well as ciliary interactions with the actin cytoskeleton [64,66]. In addition to providing a detailed view for the sequence of events, recent studies have also uncovered numerous players and pathways that govern the intracellular pathway [49]. However, less is known about mechanistic details of the extracellular pathway. Whether these two pathways have different molecular requirements and what dictates which pathway is used by different cell types remains to be addressed in future studies. 

Cilium disassembles when cells progress into the cell cycle or upon induction of differentiation in certain cell types [49]. Cilium disassembly occurs in a biphasic manner, with the first major wave during G1 shortly after mitogen stimulation and the second wave prior to mitosis [67]. Dissection of cilium disassembly in different cell types and organisms together described the physical events that underlie primary cilium disassembly, which include resorption of the ciliary axoneme to the cell body, progressive shortening of the cilium and excision of all or parts of the cilium [49,61,67,68,69,70,71]. A recent paper by Mirvis et al. 2019 used live imaging to visualize different steps of cilium disassembly in kidney epithelial cells, which uncovered heterogeneity in its dynamics and mechanisms [72]. Specifically, they proposed that cilium disassembly involves a tunable decision between two different events: ciliary resorption, in which axoneme depolymerizes and ciliary contents are incorporated into the cell; and rapid deciliation, in which the whole cilium is shed from the cell surface. Whether and to what extent this heterogeneity is shared by different cell types should be addressed in future studies. The heterogeneity among the physical events that underlie cilium disassembly is also reflected at the level of its molecular players. Among these players are the microtubule-associated proteins such as tubulin deacetylase HDAC6; severing protein katanin; depolymerizing kinesins Kif2a and Kif24; the regulatory proteins such as Aurora A, APC-Cdc20, Nek2A and the Wnt5a-CK1e-Dvl2-Plk1 pathway; dynein-binding proteins Nde1 and Tctex1 as well as the scaffolding protein HEF1/NEDD9 [67,71,73,74,75,76,77,78,79,80]. Future studies are required to unravel the mechanistic details of how these different players work together during cilium disassembly. 

### 1.3. Centriolar Satellites

Centriolar satellites are 70–100 nm membrane-less granules that localize around centrosomes and cilia (Figure 1) [2,3]. Although they are considered as the third component of the mammalian centrosome/cilium complex, less is understood about their structure, function and regulation as compared to centrosomes and cilium. Satellites are dynamic structures that exhibit both molecular motor and microtubule-dependent bimodal motility and diffusive motility [81,82]. Their dynamic behavior is important both for their cellular distribution pattern and functions in protein targeting. Assembly of satellite granules requires pericentriolar material 1 (PCM1), the molecular marker for satellites that interacts with over 200 proteins [83,84]. The satellite proteome is highly enriched in centrosome proteins and includes proteins mutated in primary microcephaly and ciliopathies. These lines of evidence confirm the tight regulatory and functional relationship of satellites with centrosomes and cilia. Consistently, satellites have emerged as key regulators of cilium assembly and maintenance, Hedgehog signaling and mitotic progression [3,85,86,87]. Recent studies aimed at dissecting satellite composition, dynamics and functions have unraveled regulation of centrosomal and ciliary protein abundance as the major mechanism by which satellites mediate their functions [2,3]. Satellites were proposed to regulate protein abundance in three different ways: 1) active transport of proteins towards and away from centrosomes and cilium, 2) sequestration of proteins at the satellites to limit their centrosomal and ciliary recruitment, 3) proteostatic regulation. Although research so far has focused on elucidating centrosomal and ciliary functions of satellites, several lines of evidence suggest possible functions outside centrosomes and cilia [2,83,84]. First, satellites are present in specialized cell types that lack centrioles. Second, satellite interactome revealed interactions with P-bodies and components of Hippo and cytokine signaling pathways. 

The cell cycle dynamics of centriolar satellites are significantly different than those of centrosomes and cilia. Among the major differences are the lack of precise number control, the liquid-like behavior and compositional and structural heterogeneity of satellite granules [2,88]. In the majority of cell types, satellites mostly cluster around centrosomes and to a less extent localize throughout the cytoplasm. As cells enter mitosis, satellites first concentrate at spindle poles and then undergo dissolution that redistributes satellite residents throughout the cytoplasm. The kinase activity of the dual-specificity kinase DYRK3 is required for the mitotic dissolution of satellites [89]. Upon mitotic exit, DYRK3 is degraded following its APC/C-mediated ubiquitination and thereby satellites reassemble and cluster around centrosomes [89]. Although the functional significance of the mitotic dissolution of satellites is not known, recent studies suggest that it might contribute to satellite functions during mitotic progression [85]. Future studies are required to elucidate the mechanisms that regulate satellite assembly, remodeling and disassembly as well as their number control.

## 2. Challenges in Mapping the Interactions at the Centrosome/Cilium Complex

The key to understanding the structure, function and dynamic regulation of the centrosome/cilium complex is the identification of its parts list and detailed characterization of how these parts come together to assemble and maintain functional centrosomes, cilium and centriolar satellites. Decades of research used structural, biochemical, cellular, proteomic and bioinformatic approaches and identified many proteins that reside at the centrosome/cilium complex. Although the cellular and organismal functions of a wide range of these proteins have been identified in targeted studies, our understanding of the interactions between these components is limited due to the inherent limitations in probing the structure of the centrosome/cilium complex. Notably, technological breakthroughs in spatiotemporal proteomic mapping, super-resolution imaging and structural biology have recently emerged as powerful tools to study the biology of the centrosome/cilium complex. In this section, we will first describe the structural, numerical and temporal challenges associated with mapping the interactions at the centrosome/cilium complex and then explain how and to what extent proximity-based labeling methods have overcome them.

### 2.1. Structural and Numerical Challenges

A major challenge for studying interactions at the centrosome is its largely insoluble nature. Although they are not bounded by membranes, centrosomes are highly structured and stable macromolecular complexes, and thus are not solubilized in the buffers used for standard biochemistry approaches such as immunoprecipitation. In fact, harsh treatment of purified centrosomes with high salt and urea concentrations does not disrupt the centriole structure and leaves an insoluble non-functional protein matrix, termed the centromatrix, associated with it [90,91,92]. Of note, the centromatrix regains its microtubule nucleation potential and parthenogenetic activity when treated with cytoplasmic extracts [91,92]. The insolubility of the centrosomes and cilia can be attributed in part to the hydrophobic interactions among its components and the highly modified microtubules of the centrioles and the ciliary axoneme (i.e., acetylation, glutamylation) [93,94,95]. A large scale bioinformatic study determined the structural properties of centrosome proteins and showed that they tend to be larger, widely modified (i.e., phosphorylation) and enriched in coiled-coil domains and disordered regions than generic human proteins [94,95]. Among the 120 full-length centrosome proteins tested, only 39 of them were soluble in expression experiments [94]. Of note, the highly modular and insoluble nature of centrosome proteins contributes to the highly ordered structure of the centrosome and its function as a scaffold. 

Another inherent limitation in studying centrosomes and primary cilium at the biochemical level is a numerical one as most cells have only one centrosome and one cilium. Additionally, the centrosomal and ciliary pools of most proteins are of lower abundance than their cytoplasmic pool. Therefore, preparing sufficient starting material for biochemical fractionation and affinity purification experiments has been a daunting task for the biochemical, proteomics and structural studies of the centrosomes and cilia. To overcome the abundance problem, proteomics of centrosomes and cilia have mostly been performed using ciliated organisms or specialized epithelia that contain hundreds of basal bodies and motile cilia [96,97,98,99,100]. Although these studies provided important insight into the composition of motile cilia, they were limited in unveiling the unique complexities of the primary cilium. As for centriolar satellites, the scale is not a limitation as the number of satellite granules varies between 300 to 500 per cell in different cell types [88].

### 2.2. Temporal Challenges

As we reviewed in the previous section, the structure and function of the centrosome/cilium complex are dynamically altered in response to different stimuli such as cell cycle cues and environmental stressors [2,61,101]. Its context-dependent adaptations require spatiotemporal modulation in the composition of centrosomes, cilium and centriolar satellites as well as posttranslational modifications of their resident proteins. As such, defining the spatial and temporal interaction maps within the centrosome/cilium complex is an essential step towards dissecting the mechanisms that underlie its diverse functions. However, the maps generated using traditional approaches were limited in the identification of the weak and transient interactions associated with the dynamic structural and functional changes of the centrosome/cilium complex. What makes these interactome studies challenging in the context of the centrosome/cilium complex is the extensive inter-organelle communication between its compartments, which hampers the identification of compartment-specific interactions. For example, about 50% of the centrosome proteome overlaps with the satellite proteome and most microtubule-associated proteins that localize to the centrosome/cilium complex are also part of cytoplasmic and mitotic microtubules [83,84].

## 3. Protein–Protein Interactions and Methods for Their Identification

The majority of proteins encoded by the human genome carry out their functions in complexes rather than as individual entities [102]. Accordingly, protein–protein interactions (PPIs) play critical roles in biological processes and thereby, unraveling the molecular underpinnings of cellular structure and function necessitates identification of the PPIs. One way of classifying PPIs is based on the proximity of the interaction: “direct” PPI if the interaction surfaces of two proteins contact with each other; “indirect” PPI if proteins are in a complex and interact via intermediate proteins. Moreover, PPIs are also referred by their lifetime of interaction, namely as “permanent” if proteins form a stable complex or “transient” if proteins interact only under specific biological contexts for a short duration of time.

To date, numerous PPI detection methods have been developed and applied in different contexts [102,103,104,105,106]. These methods are categorized into in vivo, in vitro and in silico methods, each of which has its own strengths and weaknesses. The in vivo methods include protocols performed in an organism such as yeast two-hybrid (Y2H) and synthetic lethality assays, fluorescence resonance energy transfer (FRET), biomolecular fluorescence complementation (BiFC) and proximity-labeling. In in vitro methods, protocols are performed outside an organism, such as tandem affinity purification, affinity chromatography, co-immunoprecipitation, protein arrays, X-ray crystallography and NMR spectroscopy. Finally, sequence-, structure- and gene expression-based in silico approaches are performed on a computer or by computer simulation.

Undoubtedly, components and interactions of the centrosome/cilium complex have been unveiled by a combination of these approaches over the years. These studies together led to the identification of a nearly complete parts list for the “centrosome” and partial ones for “centriolar satellites” and “cilium”. For example, the centrosome and cilium proteome identified by combining their biochemical fractionation with protein correlation profiling still serve as powerful resources [107,108]. However, the unique structural and functional complexities of the centrosome/cilium complex pose challenges in defining the spatial and temporal interactions among its components, which could not have been addressed by traditional approaches. Here, we describe and compare current methods of proximity labeling approaches and discuss their advantages and limitations for spatiotemporal mapping of interactions at the centrosome/cilium complex.

## 4. Proximity-Based Labeling Methods

**Figure 3:**
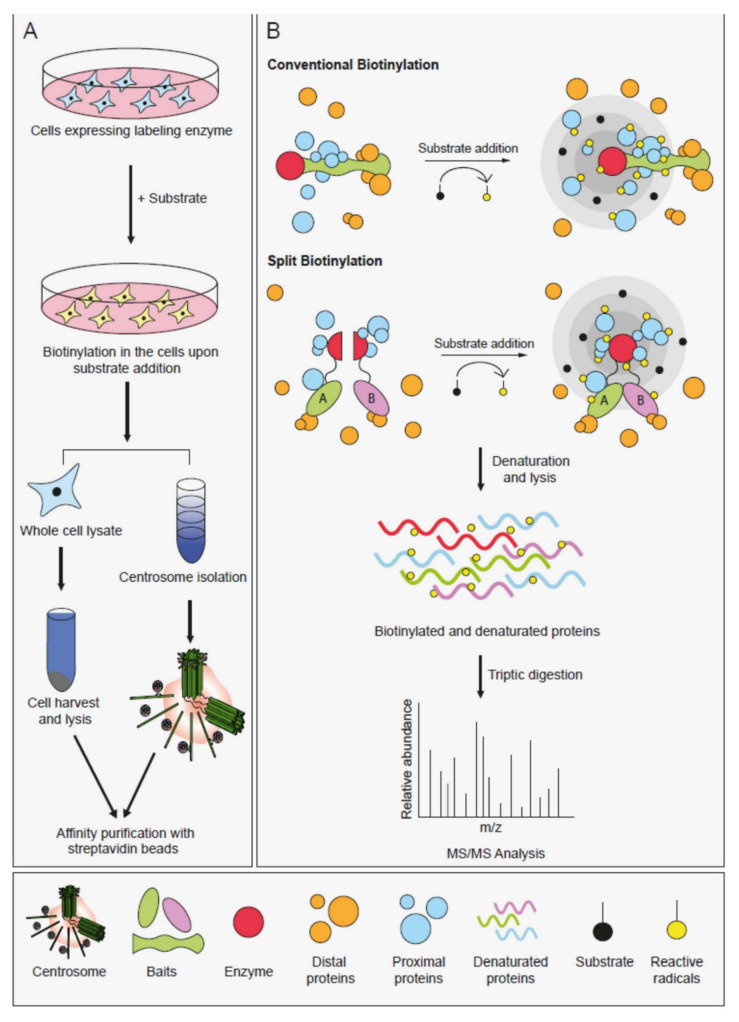
Overview of the proximity-based labeling techniques and their application to the centrosome. (**A**) Workflow of the application of proximity-based labeling to centrosomes. Cells that express proteins fused to proximity-based labeling enzymes are incubated with the labeling substrates (biotin or biotin-phenol). After biotinylation, cells are harvested and processed for the identification of biotinylated proteins in two different ways. First, centrosomes are enriched by sucrose gradients and the enriched fractions are solubilized under denaturing conditions. Second, cells are solubilized under denaturing conditions. Following lysis, the biotinylated proteins are captured by streptavidin beads and analyzed by mass spectrometry. (**B**) Schematic representation of conventional and split proximity-based labeling methods. The red circle shows the labeling enzyme that promiscuously biotinylates neighboring proteins. In the split-labeling methods, N- and C-terminal fragments of the labeling enzymes are fused to the two baits. As with protein-fragment complementation assays, the activity of the labeling enzyme is restored if the two baits associate. When substrate (biotin or biotin-phenol) is added to cells expressing enzyme fusions, the enzyme generates reactive radicals that bind to the proximal proteins (shown in blue) in the close vicinity (radius around 10 nm). The proteins that are outside of the proximity labeling radius (shown in orange) are not biotinylated. Following labeling, cells are harvested and lysed under denaturing conditions. Biotinylated proteins are captured with streptavidin beads and analyzed by mass spectrometry.

Proximity-based labeling methods have emerged as powerful tools for detecting PPIs in living cells [109,110,111]. Importantly, they overcome a number of challenges posed by conventional affinity purification methods. All proximity-based methods utilize promiscuous labeling enzymes that generate reactive radicals from either biotin or a phenolic biotin derivative. When fused to the protein of interest and expressed in a relevant biological setting, proximal proteins are covalently labeled by biotinylation and affinity purified by streptavidin pulldown following cell lysis. Subsequent mass spectrometry-based identification of the biotinylated proteins reveals the proximity landscape of the proteins of interest (Figure 3).

Since the development of the first proximity-based labeling enzyme in 2012, the field of proximity proteomics has advanced very rapidly through the development of new enzymes engineered to have a higher spatial and temporal resolution (Table 1) [110,112]. Proximity-based labeling enzymes are categorized into two main groups: biotin-based and peroxidase-based enzymes. Biotin-based proximity labeling enzymes include Biotin Identification (BioID), BioID2, BASU, TurboID and miniTurbo. The first proximity labeling enzyme developed was BioID, which is the R118G mutant of *Escherichia coli* biotin ligase (BirA). BioID uses biotin and ATP as substrates to generate reactive biotinoyl-5-AMP (bioAMP), which promiscuously labels lysine residues on proximal proteins. Mapping the interactions of the nuclear pore complex identified the labeling radius of BioID as 10 nm [113]. A smaller and improved version of BioID, namely BioID2, was engineered from *A. aeolicus* biotin ligase and was shown to exhibit enhanced proximal labeling with lower concentrations of biotin [114]. The major limitation of BioID and BioID2 is their long labeling times (18-24 hr), which is a significant barrier for mapping temporal interactions. This kinetic barrier was overcome with the development of 35 kDa TurboID and 28 kDa miniTurboID enzymes engineered using yeast display-based directed evolution [115]. These enzymes efficiently biotinylate proximal proteins in 10 min and thus offer advantages in mapping dynamic interactions with high temporal resolution. Although TurboID is more active than miniTurbo, it is 25% larger in size and induces more background biotinylation.

Engineered ascorbate peroxidase (APEX or APEX2) and horseradish peroxidase (HRP) are the peroxidase-based enzymes, which catalyze the oxidation of biotin phenol into a phenoxyl radical in the presence of H_2_O_2_ and label proteins at electron-rich side chains of their surface-exposed amino acids [116]. Given that the phenoxyl radical is very short-lived with half-life less than 1 ms, biotinylation occurs within a restricted labeling radius (<20 nm). However, the requirement of H_2_O_2_ for peroxidase-based labeling is a disadvantage to these methods as H_2_O_2_ can be toxic to living cells. A major strength of peroxidase-based enzymes over BioID is the shorter time required to generate sufficient biotinylation of the proximal proteins. Therefore, ascorbate peroxidase-based approaches have higher temporal resolution and make capturing temporal snapshots of the proximity landscape of proteins.

For spatially-controllable proximity-based labeling in cells, split versions of BioID, TurboID and APEX2 enzymes were engineered to be used in protein complementation assays [117,118,119,120]. In split versions, the inactive N and C-terminal fragments of the enzymes are fused to two proteins and the enzymes regain activity when the two proteins interact (Figure 3B). Given that biotinylation is only activated at the time and place the two bait proteins interact, split proximity-based enzymes offer an advantageous characterization of highly dynamic interactions. These approaches have so far been used to monitor the composition of spatiotemporally defined protein complexes of a microRNA-induced silencing complex and heterodimeric protein phosphatase 1 (PP1) complex [118,119].

Proximity-based proteomic profiling, regardless of the labeling enzyme used, offers two main advantages that address the structural and temporal limitations of mapping the PPIs at the centrosome/cilium complex. Both advantages are consequences of the covalent biotinylation of the proteins proximal to the bait in their native environment in living cells. Due to the strong affinity between biotin and streptavidin, cells expressing proteins of interests fused to labeling enzymes can be lysed under denaturing conditions that solubilize centrosomes while preserving the information about proximal relationships. This biochemical advantage translates into the ability to access interactions at the poorly soluble cellular structures. Additionally, given that all proximal proteins are biotinylated independent of the lifetime of their interaction with the bait protein, proximity labeling methods are superior in the identification of weak and transient interaction, and thus provide unique opportunities in mapping the spatiotemporal interactome changes of the centrosome/cilium complex. As for how proximity interaction maps compare to the ones generated by affinity purification, Lambert et al. 2014 identified the interactomes of chromatin-associated complexes by proximity-based and affinity purification-based proteomics [121]. Their results showed that the two approaches identify both distinct and overlapping interactions and are complementary to each other in interactome mapping. Therefore, proximity-based proteomics should not replace traditional approaches completely, instead their power in generating spatial and temporal snapshots of protein–protein interactions in living cells should be utilized together with the diverse toolkit of PPI identification methods. 

## 5. Application of Proximity-Based Labeling Methods to the Centrosome/Cilium Complex

The BioID approach was first developed and applied to the nuclear lamina, a highly insoluble protein complex like centrosomes, cilia and centriolar satellites. Since this landmark paper by Roux et al. 2012, the BioID method has been quickly adopted by scientists studying the centrosome/cilium complex owing to its biochemical and temporal advantages over standard methods. Here, we review the studies that applied proximity-based labeling approaches to the centrosome/cilium complex, with a particular focus on the scientific advances they uncovered in specific cellular functions and compartments (Table 2 and Table 3).

### 5.1. Centrosome Biogenesis

Analogous to DNA, centriole duplication is tightly regulated to ensure centrioles duplicate precisely only once during the cell cycle. Notably, duplication and segregation of chromosomes during the cell cycle are co-regulated with the DNA duplication cycle [101]. Deregulation of the centriole duplication cycle results in numerical aberrations such as extra centrioles, which are widespread in solid tumors and contribute to tumorigenesis by inducing aneuploidy, invasive behavior and signaling defects [142]. Before proximity-based labeling was used to dissect mechanisms of centriole duplication, numerous regulators of different steps of centriole duplication cycle have already been identified by numerous approaches such as high-throughput genetic screens, affinity purification/mass spectrometry methods and yeast two hybrid experiments [18,101]. Notably, the BioID approach has so far been extensively used to generate proximity interaction maps for known centriole duplication proteins, which identified new players and mechanisms [131]. Here, we will describe these new findings by focusing on two main stages of centriole duplication: initiation and elongation (Figure 2).

Centriole duplication is initiated by the assembly of a procentriole orthogonally to the proximal end of pre-existing centriole walls. Three evolutionarily conserved proteins are essential for this step: the kinase PLK4, the scaffold protein STIL and the building block of the cartwheel SAS-6 [143]. In mammalian cells, PLK4 is recruited to the site of procentriole formation via CEP152 and CEP192, which localize to ring-like structures at the PCM [144,145]. Following its dot-like localization at the centrioles, PLK4 phosphorylates STIL to promote the recruitment of SAS-6, which homo-oligomerizes to assemble the cartwheel [146]. The feasibility of proximity mapping for probing centrosome interactions was first tested for PLK4 because it is the master regulator of centriole duplication and identification of its substrates has been challenging due to their short half-time of association [126]. The specific PLK4 proximity partners at the centrosome were identified by BioID-based labeling combined with centrosome enrichments using sucrose gradient fractionation (Figure 3A). The PLK4 proximity map identified almost all known interactors as well as previously undescribed interactors, a number of which, such as STIL, were later characterized for their regulatory relationship to PLK4 [133,147,148]. Once the power of BioID in mapping interactions at the centrosome was shown, this approach was extended to other key regulators of centriole duplication in mammalian cells including STIL, CEP152, CEP192 and CEP63 [126]. These maps identified new regulators such as CEP85, which was shown to directly bind to STIL and regulate its centriolar targeting and function in the activation of PLK4 [133]. Strikingly, these maps for the first time revealed a regulatory relationship between centriolar satellites and centriole duplication. Specifically, satellite proteins CCDC14, KIAA0753 and CCDC57 were shown to take part in centriole duplication by regulating the centrosomal targeting of CEP63 [72,126,127,149]. Moreover, BioID-based proximity mapping of the E3 ubiquitin ligase MIB1 identified CCDC14 and KIAA0753 as potential substrates, suggesting functions for MIB1 in proteostatic regulation of centrosome proteins [117]. Another new discovery revealed by the centriole duplication maps was the relationship between PLK4 and the actin cytoskeleton. PLK4 was shown to regulate cancer cell movement by phosphorylating the Arp2 subunit of the Arp2/3 complex [137]. 

Once centriole formation is initiated, procentrioles elongate throughout the S and G2 phases. Among the key regulators of this process are CPAP and Cep135, which function in the formation and stabilization of the microtubule wall; POC5, which is required for the elongation of the distal part of the centrioles and centriole caps CP110 and CEP97, which restrict centriole length [101]. The BioID approach was used to generate proximity interaction maps for CPAP, CEP120 and PPP1R35 [126,138,150]. These maps identified the CEP120-SPICE1-CPAP functional complex as positive regulators of centriole elongation [150]. Moreover, PPP1R35 was identified as a new centriole elongation factor. Specifically, the phosphatase PPP1R35 localizes to the proximal lumen of centrioles above the cartwheel, interacts with RTTN and functions in centrosomal recruitment of CPAP, SPICE1 and POC5 [138]. Collectively, these results contributed to dissecting the pathway of centriole elongation after cartwheel formation.

In addition to centrosome duplication, proximity mapping of PCM components provided new insight into mechanisms that underlie PCM organization and mitosis. For example, proximity maps generated with N- and C-terminal BioID fusions of CEP152 revealed spatially distinct interactions, consistent with the radially elongated CEP152 localization within the PCM [126]. Validation of its interaction with CDK5RAP2 suggested functions for CEP152 in PCM recruitment and centrosome organization in mammalian cells. Additionally, proximity maps of PCM proteins previously uncharacterized for their centrosomal functions, namely SLAIN2 and CCDC61, defined the centrosomal interactions of these proteins and led to the hypothesis about their functions [128,141]. Functional assays and interaction experiments showed that CCDC61 interacts with the subdistal appendage protein CEP170 and functions during spindle assembly and symmetry. Finally, the BioID approach was also used to identify proximity interactors of a key regulator of centriole disengagement, separase, which revealed interactions with both centrosome and kinetochore proteins [140].

### 5.2. Primary Cilium Biogenesis and Proteome

Primary cilium assembly, maintenance and disassembly are highly regulated processes. To date, proximity mapping has been used to mechanistically dissect these processes using both targeted and systematic approaches. As for the targeted approaches, proximity interaction maps were generated for known regulators of cilium assembly as well as proteins mutated in ciliopathies including CEP72, CCDC66, CEP104, MIB1, ARL13B, CDC14A, LUZP1 and CEP120 [122,129,130,132,134,135,136,151]. As with centriole duplication, these maps identified new functional modules required for specific steps of cilium assembly and revealed mechanisms that underlie ciliopathies. In the proximity maps generated for retinal degeneration-associated CCDC66 and Joubert syndrome-associated CEP104 and CEP120, proteins implicated in ciliogenesis were highly enriched. Consistent with these interactions, functional studies showed that CEP104 interacts with Nek1and functions in cilium length regulation, and CCDC66 interacts with the CEP290 and is required for cilium assembly [129,151]. Additionally, structural and interaction studies of CEP120 ciliopathy mutants defined its destabilization as a consequence of the mutations and showed that mutant cells were defective in their ability to ciliate [132]. Application of proximity labeling to the small ciliary GTPase ARL13B identified interactions with GEFs, GAPs and GTPase effectors [122]. Further characterization of these relationships revealed a new mechanism for its ciliary functions, in which ARL13B interacts with the Rab11 effector FIP5 and regulates axonemal glutamylation levels by promoting ciliary import of the glutamylases TTLL5 and TTLL6. Another new discovery unveiled from proximity mapping is the regulatory relationship between the actin cytoskeleton and ciliogenesis. The actin-stabilizing protein LUZP1 and its interactor SALL1 had proximity interactions with centrosome and cilium proteins [134,135,139]. Their further characterization identified them as regulators of cilium assembly and Hedgehog signaling, and suggested mechanisms that underlie the Townes–Brocks syndrome. Notably, these results defined LUZP1 as a molecular link between the actin cytoskeleton and cilium assembly and signaling. Further supporting the relationship between actin and cilium, the dual-specificity phosphatase CDC14A was shown to regulate cilium length by modulating centrosomal actin nucleation [130].

In systematic studies of the cilium, APEX(2)-catalyzed proximity labeling was used to define the ciliary proteome [123,124]. To this end, APEX(2) was fused to cilia-targeted C-terminal regions of the G-protein-coupled receptors NPHP3 or HTR6, which localize throughout the ciliary membrane but biotinylate both the ciliary membrane and the shaft [123,124]. While both studies performed in mouse kidney epithelial cells that use the extracellular pathway of ciliogenesis, Kohli et al. 2017 study additionally used mouse fibroblasts that use the intracellular pathway of ciliogenesis [123,124]. The resulting proteomes had both overlapping and distinct proteins, and thus they complemented each other. Together, these studies identified over 200 cilium proteins, which include components of the IFT and BBSome complexes, structural components, ciliary membrane proteins, as well as previously uncharacterized components. Among the new regulators and functional modules uncovered by these proteomes are the intraciliary AC6/cAMP/PKA signaling axis, where PKA regulates Hedgehog activity by phosphorylating Gli3 in the primary cilium [123] and the identification of a wide range of actin-binding proteins in the cilium [124]. Finally, APEX-based cilium also proved to be successful in quantitatively comparing how the cilium proteome is altered in IFT27^-/-^ cells and in cells treated with actin depolymerization drugs [123,124]. Taken together, proximity-based cilium proteomics brings unique potential in mapping sensitively and efficiently how the cilium proteome changes during dynamic cellular processes and in specific disease states.

### 5.3. Proximity Landscape of Centrosomes and Centriolar Satellites

In addition to dissecting particular interactions in a targeted way, proximity-based labeling was also extensively used to create spatial and temporal interactomes for different cellular compartments [110]. The systematic mapping of centrosomes and centriolar satellites significantly advanced our understanding of the mechanisms by which these membrane-less structures are assembled from their components and maintained [83,125]. As for the centrosome, proximity mapping of 58 known centrosome proteins using the BioID approach generated an interactome map that contained > 1700 unique proteins in a network of > 7000 high-confidence proximity interactions [125]. Notably, the dynamic changes of the centrosome interactome in ciliated cells were mapped using the same approach. These maps were enriched for interactions at the sub-compartments characterized for their functions in the cilium assembly (i.e., transition zone, distal appendages). The new players and relationships identified in these maps were validated by localization and interaction studies as well as functional siRNA screens. Together with the results of validation experiments, a key finding that emerged from the centrosome proximity landscape was its high interconnectivity to the centriolar satellites, which is consistent with the proximity maps generated for centriole duplication proteins.

The centriolar satellite interactome was generated by proximity mapping of 22 known centriolar satellite proteins using the BioID approach [83]. The resulting interactome consisted of 2113 high-confidence proximity interactions among 660 unique proteins. Consistent with the functions of satellites in centrosome and cilium-related processes, about 50% of the centrosome proteome was shared with the satellite interactome. Despite this intimate molecular relationship, the satellite proximity interactome did not significantly change in centriole-less cells, suggesting that their maintenance and composition are independent of centrioles. Finally, correlation analysis of the satellite interactome with super-resolution imaging revealed compositional heterogeneity among satellite granules. Undoubtedly, the satellite interactome raises as many new questions as the ones it addressed. Future studies are required to address the centriole-independent functions and mechanisms of satellites as well as the functional significance of their size and compositional heterogeneity.

## 6. Concluding Remarks and Future Perspectives

The past six years have seen tremendous advances in our understanding of the biology of the centrosome/cilium complex due to the development of new technologies in imaging, proteomics and structural biology. Specifically, the fast-growing field of proximity-based proteomics has contributed significantly to this progress by overcoming the inherent limitations in spatial and temporal proteomic profiling of the largely insoluble, highly dynamic and low abundance centrosome/cilium complex. The spatial and temporal interactomes generated for a wide range of centrosome, cilium and satellite proteins have been instrumental in revealing new players and mechanisms for how the centrosome/cilium complex is assembled, maintained and dynamically altered.

Owing to the extensive proximity interactions of centrosome proteins with centriolar satellites, these fascinating structures have received particular attention as the third component of the centrosome/cilium complex. Functional assays combined with proteomic profiling and high-resolution imaging have together identified satellites as key regulators of the biogenesis and functions of centrosomes and cilia. Although extensive regulatory crosstalk between centrosomes, cilium and satellites is evident from numerous components they share, how these three components cooperate in diverse cellular processes remains poorly understood. The presence of shared components between centrosomes, satellites and cilium complicates the demarcation of functions for each compartment. An inducible system for localized activation or inhibition of shared components or compartment-specific mapping of the interactions could help untangle these complexities. 

Spatial and temporal proximity maps of the centrosome/cilium complex have so far been generated in mammalian cells using the BioID approach in all studies, except for the APEX-based proteomic profiling of the primary cilium (Table 2). Moreover, proximity-based labeling approaches have only been performed to probe the cilium interactome but has not been extended to generate ciliary interaction maps for axonemal proteins such as ciliary MAPs or in response to extracellular stimuli. To advance our understanding of the functions and mechanisms of centrosomes, cilium and satellites in different contexts, there are two essential steps that need to be taken in future studies. First, the proximity-based approaches should be applied in biologically relevant contexts such as tissues and organisms as well as cells treated with different stimuli. For example, knock-in mouse models expressing proximity-based labeling enzyme fusions will be instrumental in illuminating their cell type and tissue-specific interactome differences. Additionally, their application in patient samples or mouse models of human disease will provide key insight into which interactions are disrupted in disease states. Second, in addition to the BioID enzyme, the recently developed labeling enzymes that have higher efficiency and shorter labeling times (i.e., TurboID, split enzymes) should be adapted to probe the spatiotemporal changes of the centrosome/cilium complex interactomes in response to extracellular stimuli such as cell cycle cues.

## Figures and Tables

**Table 1:** **Evolution of proximity-based labeling enzymes.** The table shows the comparison of biotin ligase and peroxidase-based labeling enzymes with respect to their different properties.

	Tag	Wild Type Enzymes	Source	Molecular Weight	Substrate	Half life of Radicals	Biotinylation Time
**Conventional Biotinylation**	BioID	Biotin Ligase	E. coli	35 kDa	Biotin	Minutes	18–24 h
	BioID2	Biotin Ligase	A. Aoelicus	26 kDa	Biotin	Minutes	18–24 h
	TurboID	Biotin Ligase	E. coli	35 kDa	Biotin-phenol	Minutes	10 min
	miniTurboID	Biotin Ligase	E. coli	28 kDa	Biotin	Minutes	10 min
	APEX	Ascorbate peroxidase	Soybean	27 kDa	Biotin-phenol	<1 ms	1 min
	APEX2	Ascorbate peroxidase	Soybean	27 kDa	Biotin-phenol	<1 ms	1 min
**Split Biotinylation**	Split-BioID	Biotin Ligase	E. coli	35 kDa	Biotin	Minutes	18–24 h
	Split-TurboID	Biotin Ligase	E. coli	35 kDa	Biotin	Minutes	10 minutes
	Split-APEX	Ascorbate peroxidase	Soybean	27 kDa	Biotin-phenol	<1 ms	1 min

**Table 2:** Applications of the proximity-based labeling approaches that probed the structure and function of the centrosome/cilium complex. The table lists the bait proteins used in different proximity-based labeling methods. The following cell lines were utilized in generating their proximity interaction maps: Human embryonic kidney 293 (HEK293), Human hTERT-immortalized retinal pigmental epithelial cells (RPE1), Human renal cortical tubular epithelial cells (RCTE), Human bone osteosarcoma cells (U2OS), Mouse inner medullary collecting duct 3 (IMCD3), Mouse fibroblast cells (NIH3T3). Kidney and lung epithelial cells use the extracellular ciliogenesis pathway, whereas retinal pigmental epithelial cells and fibroblasts use the intracellular ciliogenesis pathway. Protein function information was derived from UniProt.

Tag	Protein Name	Function	Localization					Cell Line	Reference
			Centrosome	CS	Cilia	Spindle	Nucleus		
**APEX**	**ARL13B**	GTP binding/Ciliogenesis	-	-	+	-	-	RCTE	He et al., 2018 [122]
	**NPHP3(1–203)**	Ciliary membrane receptor	-	-	+	-	-	IMCD3	Mick et al., 2015 [123]
**APEX2**	**HTR6**	Ciliary membrane receptor	-	-	+	-	-	IMCD3 NIH3T3	Kohli et al., 2017 [124]
**BirA**	**AHI1**	Ciliogenesis and vesicle trafficking	+	-	+	-	-	HEK293	Gupta et al., 2015 [125]
	**B9D1**	Transition zone component/Hedgehog signalling	-	-	+	-	-	HEK293	Gupta et al., 2015 [125]
	**B9D2**	Transition zone component/Ciliogenesis	-	-	+	-	+	HEK293	Gupta et al., 2015 [125]
	**BBS4**	Ciliogenesis and ciliary sorting	+	+	+	-	-	HEK293	Gheiratmand et al., 2019 [83]
	**C11orf49**	Unknown	n/a	+	n/a	n/a	n/a	HEK293	Gheiratmand et al., 2019 [83]
	**CC2D2A**	Transition zone component/Hedgehog signalling	-	-	+	-	-	HEK293	Gupta et al., 2015 [125]
	**CCDC11**	Ciliary Beating/Ciliogenesis	-	+	+	-	-	HEK293	Gheiratmand et al., 2019 [83]
	**CCDC112**	Unknown		+				HEK293	Gheiratmand et al., 2019 [83]
	**CCDC13**	Primary cilium formation/Localization of BBS4 to cilia	-	+	+	-	-	HEK293	Gheiratmand et al., 2019 [83]
	**CCDC14**	Centriole duplication	-	+	-	-	-	HEK293	Fırat-Karalar et al., 2014 [126] Gheiratmand et al., 2019 [83]
	**CCDC18**	Unknown	n/a	+	n/a	n/a	n/a	HEK293	Gheiratmand et al., 2019 [83]
	**CCDC57**	Centriole duplication	+	+	-	+	-	HEK293	Gurkaslar et al., 2020 [127]
	**CCDC61**	Spindle assembly	+	-	-	-	-	HEK293	Barenz et al., 2018 [128]
	**CCDC66**	Ciliogenesis/Retina morphogenesis and homeostasis	+	+	+	-	-	HEK293	Conkar et al., 2017 [129] Gheiratmand et al., 2019 [83]
	**CCP110**	Centriole length regulation/Negative regulator of ciliogenesis	+	-	+	-	-	HEK293	Gupta et al., 2015 [125]
	**CDC14A**	Cilium length regulation	+	-	-	+	-	RPE1	Uddin et al., 2019 [130]
	**CENPJ/CPAP**	Centriole duplication/Centriole elongation	+	-	-	-	-	HEK293	Fırat-Karalar et al., 2014 [126] Gupta et al., 2015 [125]
	**CEP104**	Ciliogenesis and cilium length regulation	+	-	+	+	-	HEK293	Gupta et al., 2015 [131]
	**CEP120**	Centriole Duplication and Ciliogenesis	+	-	-	-	-	HEK293	Gupta et al., 2015 [131]
	**CEP120 (C2B)**	Centriole Duplication and Ciliogenesis	+	-	-	-	-	HEK293	Joseph et al., 2018 [132]
	**CEP128**	Subdistal appendages/TGFbeta signaling	+	-	-	+	-	HEK293	Gupta et al., 2015 [131]
	**CEP131**	Ciliogenesis/Spindle pole integrity	+	+	+	-	-	HEK293	Gheiratmand et al., 2019 [83]
	**CEP135**	Centriole duplication/elongation	+	-	-	-	-	HEK293	Gupta et al., 2015 [125]
	**CEP152**	Centriole duplication	+	-	-	-	-	HEK293	Fırat-Karalar et al., 2014 [126] Gupta et al., 2015 [125] Liu et al., 2018 [133]
	**CEP162**	Transition zone assembly	+	-	+	+	+	HEK293	Gupta et al., 2015 [125]
	**CEP164**	Primary cilium docking	+	-	-	-	+	HEK293	Gupta et al., 2015 [125]
	**CEP170**	Subdistal appendage assembly	+	-	-	+	-	HEK293	Gupta et al., 2015 [125]
	**CEP19**	Recruitment of ciliary vesicles	+	-	+	+	-	HEK293	Gupta et al., 2015 [125]
	**CEP192**	Centriole duplication/centrosome maturation/spindle pole assembly	+	+	-	-	-	HEK293	Fırat-Karalar et al., 2014 [126] Liu et al., 2018 [133]
	**CEP290**	Transition zone component/early ciliogenesis/ciliary targeting of cargos	+	+	+	-	+	HEK293	Gupta et al., 2015 [125]
	**CEP44**	Centriole to centrosome conversion	+	-	-	+	-	HEK293	Gupta et al., 2015 [125]
	**CEP63**	Centriole duplication/Spindle assembly	+	+	-	-	-	HEK293	Fırat-Karalar et al., 2014 [126] Gupta et al., 2015 [125] Liu et al., 2018 [133] Gheiratmand et al., 2019 [83]
	**CEP72**	Centriole duplication/spindle pole assembly	+	+	-	-	-	HEK293	Conkar et al., 2017 [129] Gheiratmand et al., 2019 [83]
	**CEP83**	Distal appendages/Ciliogenesis	+	-	-	-	-	HEK293	Gupta et al., 2015 [125]
	**CEP85**	Negative regulator of centrosome integrity	+	-	-	+	+	HEK293	Liu et al., 2018 [133]
	**CEP89**	Ciliogenesis and regulation	+	-	-	+	-	HEK293	Gupta et al., 2015 [125]
	**CEP97**	Negative regulator of ciliogenesis	+	-	-	-	-	HEK293	Gupta et al., 2015 [125]
	**CETN2**	Centriole duplication	+	-	-	+	-	HEK293	Gupta et al., 2015 [125]
**BirA**	**CNTRL**	Cell cycle and cytokinesis	+	-	-	-	-	HEK293	Gupta et al., 2015 [125]
	**CNTROB**	Centriole Duplication	+	-	-	-	-	HEK293	Gupta et al., 2015 [125]
	**DCTN1**	Dynein-mediated transport	+	-	-	+	+	HEK293	Gupta et al., 2015 [125]
	**DYNLT1**	Retrograde transport	-	-	+	+	-	HEK293	Gupta et al., 2015 [125]
	**EVC2**	Hedgehog signalling	-	-	+	-	+	HEK293	Gupta et al., 2015 [125]
	**FBF1**	Transition fiber/Cilium content regulation	+	-	-	+	-	HEK293	Gupta et al., 2015 [125]
	**FGFR1OP**	Cell cycle/Ciliogenesis	+	-	+	-	-	HEK293	Gupta et al., 2015 [125]
	**FOPNL**	Cilium biogenesis/PLK1 recruitment to centrosome	+	+	+	-	-	HEK293	Gheiratmand et al., 2019 [83]
	**KIAA0753**	Centriole Duplication	+	+	-	-	-	HEK293	Gupta et al., 2015 [125]
	**LCA5**	Intraflagellar protein transport	+	-	+	-	-	HEK293	Gupta et al., 2015 [125]
	**LUZP1**	Actin-Stabilizing protein/Ciliogenesis/Hedgehog signaling	-	-	-	-	+	HEK293	Bozal-Basterra et al., 2019 [134] Goncalves et al., 2019 [135]
	**MIB1**	Ubiquitination/Ciliogenesis	-	+	-	-	-	HEK293	Gheiratmand et al., 2019 [83] Dho et al., 2019 [136]
	**MKS1**	Transition zone regulation	+	-	+	-	-	HEK293	Gupta et al., 2015 [125]
	**NEK8**	Protein targeting to cilium	+	-	+	-	-	HEK293	Gupta et al., 2015 [125]
	**NIN**	Microtubule (-) end binding protein	+	-	-	-	-	HEK293	Gupta et al., 2015 [125]
	**NINL**	Microtubule organization	+	-	-	-	-	HEK293	Gupta et al., 2015 [125]
	**NPHP1**	Spermatogenesis/Epithelial cell polarity	-	-	+	-	-	HEK293	Gupta et al., 2015 [125]
	**NPHP4**	Ciliary trafficking	+	-	+	-	+	HEK293	Gupta et al., 2015 [125]
	**ODF2**	Microtubule organization	+	-	+	+	-	HEK293	Gupta et al., 2015 [125]
	**OFD1**	Ciliogenesis/Centriole length control	+	+	+	-	+	HEK293	Gupta et al., 2015 [125] Gheiratmand et al., 2019 [83]
	**PCM1**	Molecular marker for centriolar satellites/Ciliogenesis/Hedgehog signaling	+	+	+	-	-	HEK293	Gupta et al., 2015 [125] Gheiratmand et al., 2019 [83] Aydin et al., 2020 [85]
	**PIBF1**	Ciliogenesis/Spindle pole integrity	+	+	-	-	+	HEK293	Gheiratmand et al., 2019 [83]
	**PLK4**	Centriole duplication/Cell migration	+	-	-	-	+	HEK293	Fırat-Karalar et al., 2014 [126] Kazazian et al.,2016 [137]
	**POC1A**	Centriole stability	+	-	+	+	-	HEK293	Gupta et al., 2015 [125]
	**POC1B**	Centriole stability	+	-	+	+	-	HEK293	Gupta et al., 2015 [125]
	**POC5**	Centriole stability	+	-	-	-	-	HEK293	Gupta et al., 2015 [125]
	**PPP1R35**	Centriole elongation	+	-	-	-	-	HEK293	Sydor et al., 2018 [138]
	**RPGR**	Ciliogenesis/Photoreceptor integrity	+	-	+	-	-	HEK293	Gupta et al., 2015 [125]
	**RPGRIP1**	Photoreceptor morphogenesis/survival	-	-	+	-	-	HEK293	Gupta et al., 2015 [125]
	**RPGRIP1L**	Transition zone regulation	+	-	+	-	-	HEK293	Gupta et al., 2015 [125]
	**SALL1**	Transcriptional repressor in organogenesis	-	-	-	-	+	HEK293	Bozal-Basterra et al., 2018 [139]
	**SASS6**	Cartwheel formation during procentriole assembly	+	-	-	-	-	HEK293	Gupta et al., 2015 [125] Liu et al., 2018 [133]
	**SCLT1**	Distal appendage assembly	+	-	-	-	-	HEK293	Gupta et al., 2015 [125]
	**SEPARASE**	Centriole disengagement/Sister chromatid separation	+	-	-	-	+	U2OS	Agircan et al., 2016 [140]
	**SLAIN2**	Microtubule (+) end binding						HEK293	Fırat-Karalar, 2020 [141]
	**SPICE1**	Chromosome segregation/Centriole elongation	+	-	-	+	-	HEK293	Gupta et al., 2015 [125]
	**SSX2IP**	Centrosome maturation	-	+	+	-	+	HEK293	Gupta et al., 2015 [125] Gheiratmand et al., 2019 [83]
	**STIL**	Centriole duplication	+	-	-	-	-	HEK293	Gupta et al., 2015 [125] Liu et al., 2018 [133]
	**TBC1D31**	Unknown	+	+	-	-	-	HEK293	Gheiratmand et al., 2019 [83]
	**TCTN1**	Transition zone regulation	-	-	+	-	-	HEK293	Gupta et al., 2015 [125]
	**TCTN2**	Transition zone regulation	-	-	+	-	-	HEK293	Gupta et al., 2015 [125]
	**TCTN3**	Transition zone regulation	-	-	+	-	-	HEK293	Gupta et al., 2015 [125]
	**TEX9**	Ciliogenesis		+				HEK293	Gheiratmand et al., 2019 [83]
	**TMEM17**	Transition zone regulation	-	-	+	-	-	HEK293	Gupta et al., 2015 [125]
	**TMEM216**	Transition zone regulation	-	-	+	-	-	HEK293	Gupta et al., 2015 [125]
	**TMEM67**	Transition zone regulation	-	-	+	-	-	HEK293	Gupta et al., 2015 [125]
	**WRAP73**	Spindle pole anchoring	+	+	-	-	-	HEK293	Gheiratmand et al., 2019 [83]

**Table 3:** Biological insight gained from the application of proximity-based labeling approaches to the centrosome/cilium complex. Major findings were categorized in the context of centrosome biogenesis, mitosis, primary cilium biogenesis and compartment proteome.

Biological Process		New Findings
**Centrosome Biogenesis**	Centriole Duplication: Initiation	Identification of new duplication factors including CEP85 and STIL
		The regulatory role of centriolar satellites during initiation of centriole duplication
		Interaction of CEP152 and CDK5RAP2 in PCM recruitment
		Requiriement for MIB1 during centriole duplication
	Centriole Duplication: Elongation	Identification of CEP120-SPICE1-CPAP functional complex
		Identification of PPP1R35 as a new centriole elongation factor
	Centriole Disengament	Identification of Separase proximity interactors
	Actin Cytoskeleton	Phosphorylation of Arp2 by PLK4 for cancer cell movement
Mitosis	Spindle Assembly	Interaction of CCDC61 and CEP170 in spindle formation
Primary Cilium Biogenesis	Cilium Assembly	CCDC66 and CEP290 interaction for cilium assembly
		Involvement of CEP120 in cilium assembly
		Role of actin stabilizing protein LUZP1 in cilium assembly
	Cilium Length	Requirement of CEP104 and NEK1 interaction for cilium length regulation
		Cilia length regulation by CDC14A, which mediates centrosomal actin nucletion
	Ciliary Functions	Rab11 and ARL13B interaction in axonemal glutamylation
		Requirement for TTLL5 and TTLL6 glutamylases
		Identification of AC6/cAMP/PKA signaling module
Compartment Proteome	Centrosome	Identification of <1700 unique proteins in a netwrok of <7000 interactors
	Centriolar Satellites	Highly overlapped interactome in centrosome and satellites
		Identification of 2113 high confidance interactors among 660 unique proteins
	Cilium	Identification of over 200 proteins

## Abbreviations

PCM	pericentriolar material
MTOC	microtubule organizing center
DAP	distal appendage protein
sDAP	subdistal appendage protein
U-ExM	Ultrastructure Expansion Microscopy
STORM	Stochastic Optical Reconstruction Microscopy
NPHP	nephronophthisis
MKS	Meckel-Gruber Syndrome
JBTS	Joubert syndrome
GAP	GTPase-activating protein
GEF	Guanine nucleotide exchange factor
AURKA	Aurora kinase A
Nek2A	NIMA related kinase 2A
GPCR	G protein-coupled receptor
TTLL	Tubulin tyrosine ligase
BioID	Biotin Identification
APEX	Ascorbate Peroxidase
HTR6	5-hydroxytryptamine receptor 6
IFT	Intraflagellar Transport
BBS	Bardet-Biedl Syndrome
